# Wissen schafft Gesundheit: Das Programm „Fit in Gesundheitsfragen“ zur Stärkung der Gesundheitskompetenz von Schülerinnen und Schülern

**DOI:** 10.1007/s00103-022-03549-4

**Published:** 2022-06-03

**Authors:** Ulrike Koller, Birgit Siepmann, Verena Braun, Julia Geulen, Karen Herold, Karin Greulich-Bode, Birgit Hiller, Susanne Weg-Remers

**Affiliations:** 1 Abteilung Kommunikation, Helmholtz Zentrum München – Deutsches Forschungszentrum für Gesundheit und Umwelt GmbH, Ingolstädter Landstr. 1, 85764 Neuherberg, Deutschland; 2 Krebsinformationsdienst, Deutsches Krebsforschungszentrum, Heidelberg, Deutschland

**Keywords:** Gesundheitsförderung, Unterrichtsmaterial, Prävention, Diabetes mellitus, Krebs, Health promotion, Teaching material, Prevention, Diabetes mellitus, Cancer

## Abstract

In Deutschland weist mehr als die Hälfte der Bevölkerung eine geringe Gesundheitskompetenz auf. Diese Menschen haben Schwierigkeiten, gesundheitsrelevante Informationen zu finden, einzuordnen und anzuwenden. Unter ihnen sind auch viele junge Menschen, was den Stellenwert früher Interventionen zur Förderung der Gesundheitskompetenz verdeutlicht.

Das Programm „Fit in Gesundheitsfragen“ der Gesundheitsinformationsdienste vom Forschungszentrum Helmholtz Munich und dem Deutschen Krebsforschungszentrum hat zum Ziel, einen Beitrag zur Förderung verschiedener Dimensionen von Gesundheitskompetenz bei Kindern und Jugendlichen zu leisten. Dazu werden innovative Fortbildungsformate für Lehrkräfte und Unterrichtsmaterialien für Lernende der Sekundarstufen I und II konzipiert und begleitend evaluiert. Am Modell der Volkskrankheiten Krebs und Diabetes mellitus wird Wissen zu deren Entstehung, Prävention, Behandlung und Erforschung vermittelt. Darüber hinaus werden Wissen über das Gesundheitssystem und Materialien zur Förderung der Gesundheitskompetenz bereitgestellt. Die Evaluation erhebt Indikatoren zu Reichweite und Akzeptanz der Maßnahmen.

Seit 2018 wurden 46 Unterrichtsmaterialien und 3 Informationsschriften mit Hintergrundwissen für Lehrende veröffentlicht. Ferner wurden 50 Lehrerfortbildungen durchgeführt, an denen bis Ende 2021 rund 1600 Lehrkräfte und Multiplikatoren in Präsenz oder online teilnahmen. Sie erteilten den jeweiligen Veranstaltungen zu über 90 % sehr gute und gute Noten. Rund 80 % der Teilnehmenden gaben an, die vermittelten Themen in ihrem Unterricht aufgreifen zu wollen. Ein weiterer Ausbau des Angebots ist vorgesehen. Die Testung ausgewählter Materialien im Hinblick auf die Förderung der Gesundheitskompetenz in einer Stichprobe von Lernenden ist in Planung.

## Abstract (en)

In Germany, more than half of the population has low health literacy. These people have difficulties in finding and classifying health-related information and adapting it to their own situation. Among them are many young people, highlighting the importance of interventions early in life to promote health literacy.

This is where the “Fit in Health” program of the Health Information Services of the Helmholtz Munich Research Center and the German Cancer Research Center starts. The objective is to contribute to the promotion of different dimensions of health literacy among children and adolescents. To this end, innovative training formats for teachers and teaching materials for students at secondary levels I and II are being designed and evaluated. The major diseases cancer and diabetes mellitus are used as models to provide basic knowledge on their pathogenesis, prevention, treatment, and research. In addition, information about the structures of the healthcare system and materials to strengthen health literacy are provided. An accompanying evaluation collects indicator data on coverage and acceptance of the measures.

Since 2018, the program has published 46 teaching materials and three course readers with background knowledge for teachers. Furthermore, 50 training courses have been held, with approximately 1600 teachers and multipliers taking part in face-to-face and online events by the end of 2021. More than 90% of the participants gave the respective events very good or good evaluations. Around 80% of the participants said they would like to include the topics taught in their lessons. Further expansion of the offers is planned. Testing of a set of materials with respect to the gain of health literacy in a sample of students is currently being prepared.

## Hintergrund

Das Konzept der Gesundheitskompetenz (engl.: „health literacy“) beschreibt Wissen, Motivation und Fähigkeiten von Menschen, Gesundheitsinformationen zu finden, zu verstehen, zu bewerten und für gesundheitsbezogene Entscheidungen zur Gesundheitsförderung, Prävention oder Krankheitsbewältigung anzuwenden. Dies umfasst sowohl eine funktionale Ebene (Lesefähigkeit, Zahlenverständnis) als auch eine interaktive (Finden relevanter Informationen) und eine kritische Ebene (Bewertungskompetenz; [1]). Um neuere Entwicklungen, wie die digitale Transformation im Gesundheitswesen, abzubilden, wurde das Konzept vor Kurzem um zusätzliche Themen erweitert, etwa die „digitale Gesundheitskompetenz“ [2].

Viele Instrumente, die zur Messung der Gesundheitskompetenz entwickelt wurden, arbeiten mit Selbsteinschätzungen der Befragten. Sie sind daher eher zur Erhebung der subjektiv erlebten Passung zwischen Gesundheitsversorgung und Individuum geeignet und weniger zur Messung der individuellen Gesundheitskompetenz [1, 3]. Dennoch bieten sie einen praktikablen Ansatz für die bevölkerungsrepräsentativen und vergleichenden Health Literacy Surveys, die in Deutschland und in anderen Ländern durchgeführt wurden [4–6].

So berichten, den Ergebnissen des zweiten Health Literacy Survey (HLS-Ger-2) zufolge, 58,8 % der Menschen in Deutschland über Schwierigkeiten, gesundheitsrelevante Informationen zu finden, einzuordnen und anzuwenden [4]. Wenngleich der Anteil von Menschen mit geringer Gesundheitskompetenz laut einer Zusatzbefragung des HLS-Ger‑2 während der Coronapandemie auf 55,9 % anscheinend leicht zurückgegangen ist [7, 8], hat sich im Verlauf der letzten Jahre die Gesundheitskompetenz der Bevölkerung in Deutschland gegenüber früheren Untersuchungen tendenziell eher verschlechtert (HLS-Ger‑1 mit 54,3 % eingeschränkte Gesundheitskompetenz; [5]).

Auffällig ist in den zitierten Surveys, dass neben vulnerablen Menschen mit niedrigem Bildungsniveau, niedrigem Sozialstatus oder Migrationshintergrund sowie Älteren oder chronisch Erkrankten auch viele junge Menschen im Alter zwischen 15 bzw. 18 und 29 Jahren eine geringe Gesundheitskompetenz berichten [7, 8]. Daher gibt der Nationale Aktionsplan für Gesundheitskompetenz die Empfehlung, das Bildungssystem in die Lage zu versetzen, mit der Förderung der Gesundheitskompetenz so früh wie möglich im Leben zu beginnen [9]. Die Entwicklung und Anwendung geeigneter Instrumente zur Erhebung der Gesundheitskompetenz von Kindern und Jugendlichen und die Erprobung von Interventionen sind Gegenstand aktueller Forschung (siehe z. B. [10–14]).

Untersuchungen zeigen, dass in Abhängigkeit vom sozioökonomischen Status das Gesundheitsverhalten von Kindern und Jugendlichen schon sehr früh durch die Familie geprägt wird, etwa im Hinblick auf Ernährung, körperliche Aktivität oder Tabakkonsum [15, 16]. Gesundheitsstörungen werden vermehrt bei Heranwachsenden aus sozial benachteiligten Familien festgestellt [16]. Die Gesundheitskompetenz wird als wichtiges, modifizierbares Bindeglied zwischen einem niedrigen sozioökonomischen Status, niedrigem Bildungsniveau und dem Auftreten von Gesundheitsstörungen erkannt [17]. So kommt dem Lernort Schule eine wichtige Rolle zu, um durch eine breite Förderung verschiedener Ebenen der Gesundheitskompetenz soziale Gradienten anzugleichen [14].

Eine besondere Herausforderung liegt in der digitalen Informationsflut, die Ratsuchenden eine Fülle widersprüchlicher Gesundheitsinformationen bereitstellt. Vielen fällt es schwer, etwa während der COVID-19-Pandemie, vertrauenswürdige digitale Quellen als Basis für Gesundheitsentscheidungen zu finden [18]. Widersprüchliche Aussagen unterminieren zum einen das Vertrauen in die Gesundheitsforschung und in das Gesundheitssystem, weil unklar ist, welche Informationen verlässlich sind. Zum anderen können durch Fehlinformationen Betroffene Schaden nehmen. Auch vor dem Hintergrund der digitalen Transformation des Gesundheitswesens ist die digitale Gesundheitskompetenz, also die Fähigkeit zum eigenverantwortlichen Umgang mit digitalen Gesundheitsinformationen und -anwendungen als Schlüsselkompetenz zu sehen, die gesundheitsförderliches und präventives Verhalten unterstützt und das Handeln im Krankheitsfall erleichtert [19, 20].

Aufbauend auf diesen Erkenntnissen machen die Gesundheitsinformationsdienste von Helmholtz Munich und dem Deutschen Krebsforschungszentrum (DKFZ) mit dem Programm „Fit in Gesundheitsfragen“ einen Schritt zur Implementierung des Nationalen Aktionsplans Gesundheitskompetenz [9]. Ziel ist es, Wissens- und Fähigkeitsdefizite in den verschiedenen Dimensionen der Gesundheitskompetenz (Finden, Verstehen, Bewerten und Anwenden) in der Lebenswelt Schule zu verbessern. Mit Krebs und Diabetes mellitus, an denen in Deutschland jedes Jahr im Schnitt 500.000 bzw. 560.000 Menschen neu erkranken [21, 22], wurden Krankheitsbilder mit bevölkerungsweiter Relevanz ausgewählt. Sie sind primärpräventiven Maßnahmen zugänglich, da Lebensstil und Umweltfaktoren in ihrer Pathogenese eine bedeutende Rolle spielen (siehe z. B. [23–28]).

Das Programm besteht aus 2 Säulen: der Entwicklung und Bereitstellung von 1) innovativen Unterrichtsmaterialien für Schülerinnen und Schüler der Sekundarstufen I und II allgemeinbildender Schulen sowie 2) wissenschaftlich begleiteten Fortbildungen für Lehrende als zentrale Vermittlungsinstanz. Damit sollen evidenzbasierte Präventionsempfehlungen zu Krebs bzw. Diabetes mellitus vermittelt werden. Ferner soll Wissen zu Diagnostik und Therapie beider Erkrankungsgruppen und zu den Strukturen des Gesundheitssystems bereitgestellt werden, das die Krankheitsbewältigung unterstützen kann. Darüber hinaus sollen beide Zielgruppen – Lehrende wie Lernende – zur aktiven Informationssuche, insbesondere in digitalen Medien, befähigt und in ihrer Bewertungskompetenz gestärkt werden.

In diesem Beitrag sollen die Erstellung und Ausgestaltung der Unterrichtsmaterialien und der Fortbildung beschrieben werden. Mit der ebenfalls vorgestellten begleitenden Evaluierung werden Daten zur Nutzung und Akzeptanz der Fortbildungen und Unterrichtsmaterialien erhoben. Eine Testung ausgewählter Materialien im Hinblick auf die Förderung verschiedener Aspekte der Gesundheitskompetenz in einer Stichprobe von Schülerinnen und Schülern ist in Vorbereitung.

Grundlage für die inhaltliche Ausrichtung der Unterrichtsmaterialien und der Lehrerfortbildungen ist eine über alle Bundesländer reichende Lehrplananalyse [29], die zu Beginn des Vorhabens durchgeführt wurde. Demnach haben die Themen Diabetes mellitus und Krebs Anknüpfungspunkte in zahlreichen Unterrichtsfächern: Neben naturwissenschaftlichen Fächern wie Biologie sind das Sport, Ethik, Deutsch, Arbeitslehre, Pädagogik, Psychologie sowie gesellschaftswissenschaftliche Fächer. Als Basis für die Erstellung der Inhalte dienten die evidenzbasierten Wissensdatenbanken zu Krebs und Diabetes mellitus der Projektpartner, die nach definierten Methodiken erstellt und aktualisiert werden [30, 31].

Die folgenden Themen stehen im Fokus:

- Prävention von Krebs und Diabetes mellitus
- Biologische Prinzipien der Entstehung, Diagnose und Behandlung von Krebs und Diabetes mellitus
- Wissen zu den Strukturen des Gesundheitssystems
- Kompetenzen im Umgang mit Gesundheitsinformationen (Suchen, Finden, Bewerten), insbesondere mit digitalen Angeboten

Passend zu dem zugrunde liegenden Wissen wurden didaktische Methoden im Hinblick auf ihre Eignung zur Vermittlung der Inhalte sowie zur Förderung der interaktiven und Bewertungskompetenzen der Lernenden ausgewählt.

## Unterrichtsmaterialien

Mit den Unterrichtsmaterialien soll aktuelles, evidenzbasiertes Fachwissen vermittelt werden. Darüber hinaus sollen die Fähigkeiten der Schülerinnen und Schüler zur aktiven Informationssuche und zur Bewertung von Gesundheitsinformationen gefördert werden und sie sollen zu eigenverantwortlichen Gesundheitsentscheidungen befähigt werden.

Das Spektrum umfasst klassische und innovative Materialien – sowohl im Hinblick auf die verwendeten Medien (PDF-Dateien zum Ausdrucken oder digitale Medien) als auch auf die didaktischen Methoden. So gibt es klassische Infoblätter, Lernimpulse oder Lernzirkel mit Arbeitsblättern, die beispielsweise Lückentexte oder Arbeitsaufträge enthalten. Ergänzt wird dies durch digitale Materialien wie interaktive Grafiken, Erklärvideos und E‑Learning-Module. Damit steht den Lehrkräften ein Baukastensystem mit Materialien für insgesamt 68 Doppelstunden (Stand 28.12.2021) zur Verfügung, das sie je nach Bedarf und Möglichkeiten im Sinne des Blended Learning (integriertes Lernen) im Unterricht zum Einsatz bringen können. Eine Vielzahl an didaktischen Methoden kommt zur Anwendung, von denen einige in diesem Beitrag exemplarisch dargestellt werden.

Die Materialien sind altersgerecht für verschiedene Klassenstufen aufbereitet und berücksichtigen die Lebenswelten und soziokulturellen Bedingungen der Schülergruppen, indem sie etwa Probleme von Jugendlichen mit Diabetes mellitus im schulischen Umfeld ansprechen oder Smartphone-Apps zur Bearbeitung von Aufgaben zur Krebsprävention einsetzen. Zudem werden Elemente integriert, die soziale und kreative Fähigkeiten fördern und den Lernenden ermöglichen, Inhalte in Teamarbeit zu erarbeiten oder in systematischen Prozessen gemeinsam neue Ideen hervorzubringen. Bei vielen Materialien spielen die Recherche in digitalen Medien, die Beurteilung der Seriosität von Informationsanbietern und die Einordnung des gefundenen Wissens eine zentrale Rolle.

In die fortlaufende Entwicklung der Unterrichtsmaterialien wird Feedback von Lehrenden und Lernenden im Rahmen von Fortbildungen und Projekttagen einbezogen. Sie zeigen, dass die Lernenden gerne mit konkreten Fallbeispielen arbeiten und sich einen möglichst realitätsnahen Alltagsbezug wünschen.

Entsprechend wird im Rahmen der Entwicklung darauf geachtet, dass Schülerinnen und Schüler in den Materialien Identifikationsfiguren finden, aber auch Empathie für die Rolle von Erkrankten entwickeln können. Dazu werden neben Lerneinheiten, die primär der Wissensvermittlung dienen, auch solche entwickelt, die sich mit der Erkrankung aus Sicht von Angehörigen oder Betroffenen beschäftigen oder die Möglichkeit bieten, deren Perspektive einzunehmen. So wurde beispielsweise in der Lerneinheit „Diabetes mellitus, Digitalisierung und Design Thinking“ der Design-Thinking-Ansatz [32] verwendet. Dabei handelt es sich um eine Kreativitätsmethode, bei der es zentral ist, empathisch die Perspektive Betroffener einzunehmen und Lösungen für Alltagsprobleme neu zu denken.

Alle Materialien durchlaufen einen mehrstufigen Redaktionsprozess. Sie werden zum kostenlosen Download auf den Projektseiten www.krebsinformationsdienst.de und www.diabinfo.de angeboten [33, 34] und können für den Unterricht ausgedruckt oder digital genutzt werden.

## Fortbildungsprogramm für Lehrkräfte

Lehrkräfte werden gezielt mit wissenschaftlich begleiteten Fortbildungen angesprochen, die der Wissens- und Kompetenzvermittlung und der Einführung der Unterrichtsmaterialien dienen. Die Teilnahme ist freiwillig und kostenlos. Die Fortbildungen werden in Zusammenarbeit mit Fortbildungszentren für Lehrkräfte verschiedener Bundesländer (z. B. Bayern, Niedersachsen, Hessen, Rheinland-Pfalz) konzipiert und beworben. Darüber hinaus werden sie über Netzwerke wie dem der Helmholtz-Schülerlabore, Schulverteiler und die Bildungsserver bekannt gemacht.

Die Fortbildungen sind in der Regel zweiteilig konzipiert: Im ersten Teil wird den teilnehmenden Lehrkräften Fachwissen durch Vorträge von Forschenden aus Helmholtz Munich bzw. DKFZ zum jeweiligen Themenschwerpunkt vermittelt. Er basiert auf aktuellen Forschungsergebnissen. Im zweiten Teil werden die darauf aufbauenden Unterrichtsmaterialien vorgestellt und in ein didaktisches Konzept eingeordnet. Darüber hinaus ermöglichen die Veranstaltungen, in einem interaktiven Ansatz ein direktes Feedback zu den Materialien von den Teilnehmenden einzuholen und dieses zur weiteren Optimierung zu nutzen.

Ab März 2020 wurden pandemiebedingt sämtliche Veranstaltungen in bundesweit zugängliche, digitale Formate überführt, wodurch eine überregionale Reichweite erzielt werden konnte. Die Dauer beträgt in der Regel anderthalb bis 2 h am späten Nachmittag. Dadurch ist den Lehrenden eine niederschwellige Teilnahme von Zuhause aus möglich, ohne Freistellung vom Unterricht.

Umfassende Informationsschriften (Reader) für Lehrkräfte, die unabhängig von den Fortbildungen online zur Verfügung stehen, fassen das in den Fortbildungen vermittelte Hintergrundwissen zusammen und verweisen auf eine Auswahl zugrunde liegender Quellen.

## Evaluation

In der hier beschriebenen Projektphase soll erhoben werden, welche Reichweite das Programm insbesondere mit den Fortbildungsveranstaltungen erzielt und wie zufrieden die teilnehmenden Lehrkräfte mit der Fortbildung sind.

Hierfür wird fortlaufend die Zahl der Teilnehmenden dokumentiert und im Anschluss an jede Veranstaltung die Zufriedenheit mit der jeweiligen Fortbildung und den vorgestellten Materialien einschließlich der Nutzungsabsicht im Unterricht mit standardisierten Feedbackfragebögen [35] datenschutzkonform quantitativ erfasst. Die erhobenen Daten tragen zur Optimierung des Veranstaltungsangebots bei, vermitteln aber auch einen Eindruck über die Akzeptanz des Programms. Erhoben werden unter anderem:

- Zufriedenheit der Teilnehmenden mit dem zeitlichen und inhaltlichen Umfang der Fortbildung und der Fortbildung insgesamt,
- Beurteilungen zur Interessantheit, Verständlichkeit und Unterrichtsrelevanz der einzelnen Vorträge,
- Bewertung der vorgestellten Unterrichtsmaterialien (u. a. hinsichtlich Verständlichkeit und Umfang),
- Einschätzung, ob die Teilnehmenden für sich selbst relevante Informationen zu Krebs bzw. Diabetes mellitus erhalten haben und ob sie die behandelten Themen in ihrem Unterricht aufgreifen werden.

Ein Eindruck über die Reichweite lässt sich auch über das Monitoring der Programmwebsites gewinnen, bei dem mit den Tools Econda (für die Seite des DKFZ) bzw. Matomo (für die Seite von Helmholtz Munich) kontinuierlich die Nutzung, etwa die Zahl der Downloads der Unterrichtsmaterialien, erfasst wird.

## Ergebnisse

### Unterrichtsmaterialien

Die Unterrichtsmaterialen sind als modulares System aufgebaut, um Lehrkräften eine individuelle Zusammenstellung, abgestimmt auf die Lernbedürfnisse ihrer Schülerinnen und Schüler und den verfügbaren zeitlichen Umfang, zu ermöglichen. Entsprechend der derzeit an Schulen noch sehr unterschiedlichen technischen Möglichkeiten werden sowohl klassische als auch digitale Materialien angeboten.

Insgesamt stehen Lehrkräften derzeit (Stand 28.12.2021) 46 Unterrichtsmaterialien zum direkten Einsatz in der Schule kostenfrei zur Verfügung [33, 34]. Hintergrundwissen für Lehrende zu Krebs und Diabetes mellitus wird in 3 Informationsschriften angeboten. Die Unterrichtsmaterialien und Informationsschriften werden zum Download auf den Internetseiten der Projektpartner angeboten und über verschiedene Kanäle sowie mittels eigener Newsletter beworben. Eine Übersicht der Unterrichtsmaterialien und Reader ist in den Tab. 1, 2 und 3 zu finden. Ein weiterer Ausbau des Angebots bis 2022 und nachfolgend eine regelmäßige Prüfung und Aktualisierung der Materialien sind vorgesehen.

#### Beispiele Diabetes mellitus

In der Lerneinheit „Der Blutzuckerspiegel im Tagesverlauf“ erwerben die Schülerinnen und Schüler Wissen zum Blutzuckerspiegel im Tagesverlauf, indem sie den Protagonisten durch den Tag begleiten. In einer webbasierten Trainingseinheit „Klassenfahrt trotz Diabetes – geht das?“ erarbeiten sie gemeinsam mit einer fiktiven, an Typ-1-Diabetes erkrankten Mitschülerin, wie sich ihr Alltag verändert, wie technische Anwendungen, etwa eine Insulinpumpe, funktionieren oder wie man in einer Notsituation reagiert. Sie klären auch, ob man trotz Diabetes mellitus an einer Klassenfahrt teilnehmen kann.

In der Lerneinheit „Diabetes, Digitalisierung und Design Thinking“ entwickeln die Lernenden den Prototyp einer Gesundheits-App, mit der sie entweder Menschen mit Typ-1-Diabetes mellitus unterstützen oder präventiv gegen die Entstehung von Typ-2-Diabetes mellitus aktiv werden können. Sie diskutieren dazu mit Betroffenen und lernen Leben und Umgang mit der Erkrankung kennen und verstehen. Zugleich setzen sich die Schülerinnen und Schüler damit auseinander, ob neue Technologien einen Beitrag zur Verbesserung der Lebensumstände von Menschen mit Diabetes mellitus leisten können.

#### Beispiele Krebs

Ein „stummes“ Erklärvideo versprachlichen – mit dieser kreativen Methode erarbeiten sich Lernende in der Lerneinheit „Selbst erklärt – So entwickelt sich Krebs“ Wissen zur Krebsentstehung, zur Metastasierung sowie zu den Krebsrisikofaktoren. Der fehlende Sprechertext wird von den Schülerinnen und Schülern selbst verfasst und das Video anschließend vertont. Durch vielfältige Differenzierungsangebote lässt sich die Lerneinheit sowohl in Sekundarstufe I als auch in Sekundarstufe II einsetzen.

In der Lerneinheit „Zielgerichtete Krebstherapie“ lernen Schülerinnen und Schüler der Sekundarstufe II mithilfe eines „Mystery“^1^ die Entwicklung einer zielgerichteten Krebstherapie – des Tyrosinkinaseinhibitors Imatinib gegen die chronisch myeloische Leukämie (CML) – exemplarisch kennen. Die Herausforderung besteht darin, die Informationen der Rätselkarten korrekt in Beziehung zu setzen und so die Lösung zu finden. Sie ergibt sich aus einem zufälligen Missgeschick im Labor, das zur Identifizierung des Philadelphia-Chromosoms führte – relevant für über 90 % der an CML Erkrankten. Das „Mystery“ folgt dem Weg der Forschung über die Signaltransduktion von Krebszellen bis hin zu den daraus abgeleiteten Krebstherapien.

Die Unterrichtsmaterialien sind über die Programmwebsites [33, 34] frei zugänglich. Die Websites wurden am 15.02.2020 (Helmholtz Munich) bzw. am 16.04.2020 (Deutsches Krebsforschungszentrum) mit einem Angebot veröffentlicht, das seither kontinuierlich ausgebaut wird. Die online verfügbaren Unterrichtsmaterialien wurden seit dem Launch insgesamt rund 11.000-mal von den Websites heruntergeladen.

### Fortbildungsprogramm für Lehrkräfte

Seit Start des Programms im April 2018 wurden insgesamt 50 thematische Lehrerfortbildungen und Schulungen zur Vorstellung des Programms in Präsenz oder online durchgeführt, die bis Ende Dezember 2021 rund 1600 Lehrkräfte, Lehramtsstudierende der Carl von Ossietzky Universität Oldenburg und Multiplikatoren aus dem bayerischen Kultusministerium erreichten. Darüber hinaus wurden Materialien zu verschiedenen Themen in 8 Projekttagen gemeinsam mit Lehrkräften und rund 220 Schülerinnen und Schülern direkt erprobt.

Teilnehmende Lehrerinnen und Lehrer stammten mehrheitlich aus Gymnasien (42 %). Die Fortbildungen waren aber auch für Lehrkräfte berufsbildender Schulen interessant. Sie stellten mit 21 % die zweitgrößte Teilnehmergruppe dar, vor den Teilnehmenden aus verschiedenen anderen Schularten und Lehramtsanwärtern (18 %) sowie den Lehrenden aus der Realschule bzw. Realschule plus (11,3 %).

### Evaluation der Lehrerfortbildungen

**Figure 1:**
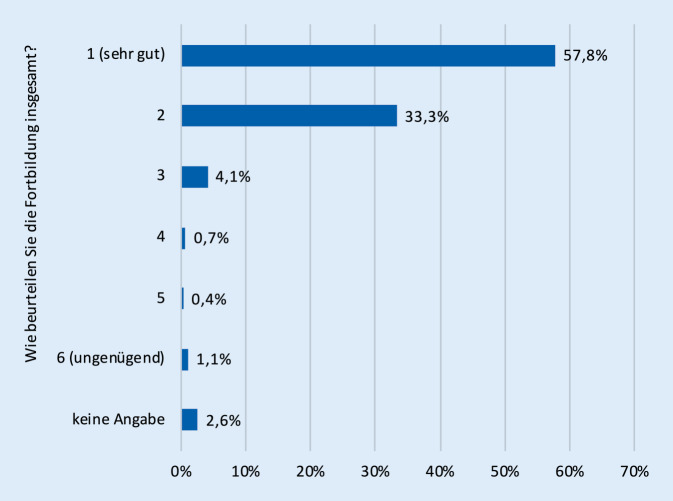

**Figure 2:**
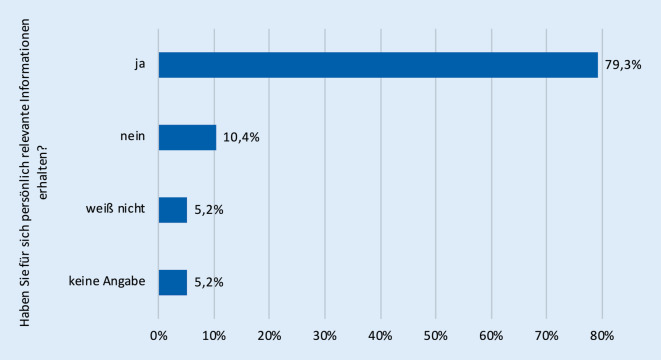

**Figure 3:**
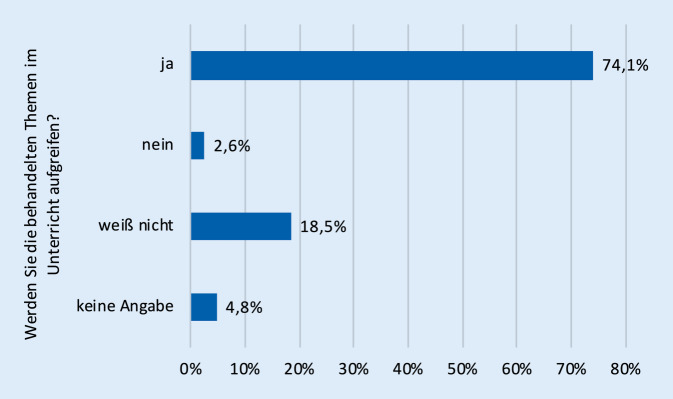

Eine Zwischenauswertung der Feedbackfragebögen zu den Fortbildungen zeigt eine überwiegend positive bis sehr positive Resonanz (Stand Dezember 2021, nur thematische Onlinefortbildungen, *n* = 910 Teilnehmende, ausgefüllte Onlinefragebögen *n* = 270, Response-Rate 30 %). Die Zufriedenheit mit den bislang durchgeführten Fortbildungen war mit über 91 % guten (33,3 %) und sehr guten (57,8 %) Bewertungen insgesamt sehr hoch (Abb. 1). Auch der persönliche Wissenszuwachs war hoch: Rund 80 % der Teilnehmenden gaben an, für sich selbst relevante Informationen zu Diabetes mellitus oder Krebs erhalten zu haben (Abb. 2). Mit ca. 74 % gab die überwiegende Mehrzahl der Lehrerinnen und Lehrer an, die vorgestellten Themen künftig in ihrem Unterricht aufgreifen zu wollen (Abb. 3). Lediglich 2,6 % schlossen dies für ihren Unterricht aus, ohne dass sich ein klarer Zusammenhang zu bestimmten Schularten feststellen ließ. 18,5 % der Umfrageteilnehmenden wussten es noch nicht bzw. machten keine Angabe (4,8 %).

Die direkten Rückmeldungen von Lehrkräften in den Veranstaltungen waren durchweg positiv. Gelobt wurde vor allem der Pool an aktuellen, kreativen und ansprechend gestalteten Unterrichtsmaterialien. Als mögliche Barriere wurde der Umfang der Lehr- und Bildungspläne der Länder genannt, der es oft nicht erlaubt, Gesundheitsthemen stärker im Unterricht zu integrieren.

## Diskussion

Die beiden großen repräsentativen Surveys [4, 5] zur Erfassung der Gesundheitskompetenz der deutschen Bevölkerung legen nahe, dass die Mehrheit der Menschen in unserem Land Schwierigkeiten erlebt, sich gesundheitsbezogene Informationen im Alltag zu erschließen und darauf beruhende „gute“ Gesundheitsentscheidungen für sich selbst zu treffen. Der daraus resultierende Handlungsbedarf hat auf der gesundheitspolitischen Ebene zur Gründung der Nationalen Allianz für Gesundheitskompetenz und zur Entwicklung des Nationalen Aktionsplans Gesundheitskompetenz geführt [9], der Maßnahmen zur Stärkung der Gesundheitskompetenz in allen Lebenswelten vorschlägt. Im Rahmen verschiedener Vorhaben wurden und werden Maßnahmen und Interventionen zur Stärkung der Gesundheitskompetenz der Bevölkerung konzipiert und implementiert [37]. Ein Fokus liegt dabei auf Personen, die besonders vulnerabel sind, etwa älteren Menschen, chronisch Kranken oder Menschen mit einem niedrigen sozioökonomischen Status, die ebenfalls häufig eine Beeinträchtigung ihrer Gesundheitskompetenz erleben.

Kinder und Jugendliche sind eine weitere relevante Zielgruppe, um ungünstige sozioökonomische Determinanten [15] durch schulische Interventionen auszugleichen und Gesundheitskompetenz früh im Leben zu fördern. Unter Einbeziehung des Lernorts Schule und der dort tätigen Lehrenden als zentrale Vermittler ist es Ziel des Programms „Fit in Gesundheitsfragen“, junge Menschen anzusprechen und in ihrer Gesundheitskompetenz, insbesondere auch im Umgang mit digitalen Angeboten, nachhaltig zu stärken. Einen besonderen Schwerpunkt setzt das Programm in der Stärkung der Bewertungskompetenz beider Zielgruppen – der Lehrenden und der Lernenden – zur Einordnung von selbst recherchierten, digitalen Gesundheitsinformationen.

Das Programm „Fit in Gesundheitsfragen“ hat seit seinem Start im April 2018 eine überregionale Bekanntheit erlangt. Angestrebt war eine jährliche Teilnehmerzahl an Fortbildungsveranstaltungen von bis zu 500 Lehrenden ab dem 2. Projektjahr. Mit 1600 Teilnehmenden bis Ende 2021, also noch vor Abschluss des 4. Projektjahrs, lag die Zahl deutlich über den Erwartungen. Die Zahl der Downloads von Unterrichtsmaterialien von den Projektwebsites ist mit 11.000 seit dem Launch ein weiterer Indikator für die Reichweite, allerdings wird die Validität der Downloadzahlen durch die geltenden Datenschutzbestimmungen teilweise eingeschränkt, die beispielsweise keine Erfassung wiederholter Downloads von einer IP-Adresse aus erlauben und Nutzern die Abschaltung von „Tracking-Cookies“ ermöglichen.

Das Konzept des Programms mit seinem Angebot an wissenschaftlich fundierten Lehrerfortbildungen in Kombination mit der Bereitstellung von innovativen und klassischen Unterrichtsmaterialien weist eine hohe Praxisnähe auf: Die Teilnehmenden berichteten interaktiv während der Veranstaltungen und per Onlinefeedbackfragebogen ganz überwiegend eine hohe bis sehr hohe Zufriedenheit mit den angebotenen Fortbildungen und Unterrichtsmaterialien, eine hohe Intention zum Aufgreifen der vermittelten Themen im Unterricht und einen sehr guten eigenen Wissenszuwachs. Die mit 30 % unter dem publizierten Durchschnitt von 34 % für Onlinebefragungen [38] liegende Response-Rate begrenzt die Aussagekraft der erhobenen Daten. Der Rücklauf wäre in Präsenzveranstaltungen wahrscheinlich höher. Diese waren jedoch unter Pandemiebedingungen nicht möglich.

Ein wesentlicher Qualitätsfaktor ist das wissenschaftliche Renommee der beiden Träger des Programms, Helmholtz Munich und Deutsches Krebsforschungszentrum, als international sichtbare Großforschungseinrichtungen. An beiden Einrichtungen existieren seit vielen Jahren Wissenstransferplattformen wie der Krebsinformationsdienst und der Diabetesinformationsdienst, der 2020 gemeinsam mit dem Deutschen Zentrum für Diabetesforschung und dem Deutschen Diabeteszentrum in Düsseldorf in das nationale Diabetesinformationsportal diabinfo.de überführt wurde. Beide Partner bieten gut etablierte Onlineportale, auf denen Fortbildungsangebote und Unterrichtsmaterialien eine ausgezeichnete Sichtbarkeit erlangen.

Sicher war es auch der COVID-19-Pandemie geschuldet, dass sich Lehrkräfte außergewöhnlich schnell neuen, innovativen Materialien sowie digitalen Fortbildungsformaten geöffnet haben [39].

Offen ist derzeit noch die Frage, ob und in welchem Maß das Programm „Fit in Gesundheitsfragen“ mit seinen Angeboten die verschiedenen Dimensionen der Gesundheitskompetenz von Schülerinnen und Schülern tatsächlich stärken kann. Um dies zu beantworten, ist in der Abschlussphase des Projekts eine Interventionsstudie in Schulklassen unter Nutzung ausgewählter Lerneinheiten vorgesehen, die gemeinsam mit Partnern aus der Wissenschaft auf Basis aktueller Forschungsarbeiten zur Erhebung der Gesundheitskompetenz von Kindern und Jugendlichen [13, 40] durchgeführt werden soll.

2 Aspekte stellen im Konzept des Programms „Fit in Gesundheitsfragen“ eine besondere Herausforderung dar: Dies betrifft zum einen die nachhaltige Verbesserung der Gesundheitskompetenz mit langfristigen Effekten auf gesundheitliche Entscheidungen, Gesundheitsverhalten und Krankheitsbewältigung. Dies mag für manche gesundheitsbezogenen Entscheidungen einfacher zu erreichen sein als für andere, z. B. eher für die Impfung gegen humane Papillomviren (HPV) zur Prävention von HPV-induzierten Tumoren als für Ernährungsfaktoren, die durch das soziale Umfeld in den verschiedenen Lebenswelten dauerhaft modifizierbar bleiben. Unklar ist zum anderen auch, ob das Programm geeignet ist, soziale Ungleichheit in der Gesundheitskompetenz abzubauen. Die Auswertung der Feedbackfragebögen zeigte, dass vor allem Lehrende aus Gymnasien an den Fortbildungen teilnahmen. Lehrkräfte von berufsbildenden Schulen stellten jedoch die zweitstärkste Gruppe dar, sodass von einem großen Interesse dieser Gruppe an den Fortbildungsthemen auszugehen ist – wenn auch überwiegend bezogen auf die spezifischen beruflichen Ausbildungsgänge.

## Fazit

Eine bildungspolitische Forderung wäre, dass Gesundheitsthemen in den Bildungsplänen der Bundesländer für die Schulen einen größeren Raum als bisher erhalten, gerade auch vor dem Hintergrund der COVID-19-Pandemie, der digitalen Transformation des Gesundheitssystems und politischen Forderungen nach einer Stärkung der Gesundheitskompetenz schon im Kindes- und Jugendalter. Dies würde Lehrende sicherlich in hohem Maße dabei unterstützen, Gesundheitsthemen im Unterricht aufzugreifen. Die positiven Reaktionen der Lehrenden auf das Programm „Fit in Gesundheitsfragen“ sprechen für ein großes Interesse an Gesundheitsthemen und für die Praxisnähe des Konzepts, das Fortbildungsangebote mit darauf aufbauenden wissenschaftlich und didaktisch aufbereiteten Unterrichtsmaterialien zu Gesundheitsthemen kombiniert.

## Anhang

**Table 1:**

Thema	Inhalte	Material/Methode
Gesundheit erhalten – Krankheit vermeiden	Wissen, aktuelle Forschung und Präventionsansätze am Beispiel Diabetes	Reader für Lehrkräfte, 26 Seiten
Diabetes mellitus – Was ist das eigentlich?	Gesamtüberblick über die Krankheit (Symptome, Diagnose); Unterschied zwischen Typ-1- und Typ-2-Diabetes	Lerneinheit
Die Bauchspeicheldrüse	Aufbau, Lage, Morphologie, Aufgaben, Entstehungsmechanismen von Typ-1-Diabetes, Botenstoff Insulin	Lerneinheit inkl. Wissensüberprüfung und Erklärvideo
Der Zuckerstoffwechsel	Beteiligte Organe u. Organsysteme, Aufbau von Kohlenhydraten, Regulation des Blutzuckerspiegels durch die Antagonisten Insulin und Glukagon	Lerneinheit
Sport und Bewegung – Was hat das mit Diabetes zu tun?	Bedeutung von Bewegung für die Gesunderhaltung des eigenen Körpers und zur Vorbeugung von Typ-2-Diabetes; altersgruppenspezifische Empfehlungen für Bewegung und Sport	E‑Learning-Einheit mit Lernspiel und Quiz
Sport und Bewegung – Was hat das mit Diabetes zu tun?	Zusammenhänge zwischen körperlicher Aktivität und Reaktionen des Körpers, Auswirkungen regelmäßiger körperlicher Belastung auf das Herz-Kreislauf-System, Bedeutung von Bewegung für die Gesunderhaltung und die Vorbeugung von Typ-2-Diabetes	Lerneinheit
Sportverhalten und Motivation	Reflexion des eigenen Sportverhaltens; Aspekte von Motivation in Bezug auf Sport; Bedeutung von Sport und Bewegung zur Prävention von Diabetes mellitus	„WOOP-Methodik“ nach G. Oettingen [36], Erklärvideo zur WOOP-Methode (keine Eigenproduktion) und Einbinden der WOOP-App
Apps zur Förderung von Bewegung und Sport nutzen	App „Actionbound“; Stärkung der medialen Kompetenzen, Nutzung digitaler Lernwerkzeuge	Lerneinheit
Gegenspieler Insulin und Glukagon	Lernspiel, das die Eigenschaften von Insulin und Glukagon sowie deren Auswirkungen auf den Blutzuckerspiegel verdeutlicht und zur Bewegung anregt	Lern- und Laufspiel für den kombinierten Unterricht Biologie und Sport
Gesund ernähren – Diabetes vorbeugen. Erkenntnisse aus eigenen Erfahrungen schöpfen	Schwierigkeiten, sich gesund zu ernähren; Wege finden, dennoch auf Gesundheit zu achten; „Nudging“ (Anstoßen gesundheitsförderlicher Verhaltensweisen); gesundheitsbewusste Entscheidungen und Empathievermögen; Unterscheidung „gesunde Ernährung“ und „Genuss“	Lerneinheit
Planspiel Zuckersteuer	Diskussion einer möglichen staatlichen Präventionsmaßnahme, Risikofaktoren für Typ-2-Diabetes, Einfluss stark zuckerhaltiger Ernährung auf die Gesundheit	Lerneinheit
Ernährung und Diabetes	Ergebnissicherung als Aufgabe in Breakout-Edu (Escape Game fürs Klassenzimmer) eingebunden	Quiz
Typ-F-Diabetes	Kennenlernen des Alltags eines Betroffenen, digitale Anwendungen bei Diabetes, Podcasts von Menschen, die an Diabetes erkrankt sind	Lerneinheit, Rollenspiel
Diabetes, Digitalisierung und Design Thinking	Krankheitsverlauf von Typ-1- und Typ-2-Diabetes; Austausch mit Betroffenen; digitale Helfer, Apps; Entwicklung eines App-Prototypen	Lerneinheit, Konzept „Design Thinking“
Diabetesforschung	Aufbau und Vorgehen in der Diabetesforschung, verschiedene Forschungsbereiche, Zuckerstoffwechsel, Insulin und Glukagon, Blutzuckermessung	Lerneinheit, interaktive Erklärgrafik
Naturwissenschaftlicher Erkenntnisweg	Aufbau und Interpretation wissenschaftlicher Arbeiten am Beispiel neuer Forschungsergebnisse aus der Diabetesforschung	Lerneinheit
Der Blutzuckerspiegel im Tagesverlauf	Auswirkungen verschiedener Lebensmittel und sportlicher Aktivität auf den Blutzuckerspiegel im Schulalltag und anderen Lebenswelten	E‑Learning-Modul
Digitale Anwendungen bei Diabetes	Methoden zur Blutzuckermessung und Blutzuckerregulation (kontinuierliche Gewebezuckermessung (CGM), Hybrid-Closed-Loop-System), Zukunft der Insulintherapie	E‑Learning-Modul
Klassenfahrt trotz Diabetes – Geht das?	Typ-1-Diabetes aus Sicht einer jungen Betroffenen, Alltag mit der Autoimmunerkrankung, Grundlagen von Typ-1-Diabetes, Sensormessung und Insulinpumpe, Anzeichen für Unterzuckerung, Hilfe im Notfall	E‑Learning-Modul
Prävention durch Sport und Bewegung	Positive Auswirkungen von Sport und Bewegung auf den Körper, Zusammenfassungen und Tipps für den Alltag, Treffen von aktivitätsbezogenen Entscheidungen, Auswirkungen auf Konzentrationsfähigkeit und Wohlbefinden	E‑Learning-Modul
Die Bauchspeicheldrüse	Aufbau und Aufgaben der Bauchspeicheldrüse, Wirkmechanismus von Insulin, Tipps zur Vorbeugung von Typ-2-Diabetes	Erklärfilm
Autoimmunerkrankung – Das Beispiel Diabetes	Mechanismen, die zum Ausbruch von Typ-1-Diabetes führen, Inselautoantikörper, Früherkennung von Typ-1-Diabetes	Erklärfilm
Das Hormon Insulin	Insulin zur Energieversorgung des Körpers, Insulinempfindlichkeit des Gehirns, Auswirkungen auf Essverhalten, Insulinmangel, Insulinresistenz, Rolle von Insulin bei der Entstehung von Diabetes, Insulinspritzen, Insulinpumpe, künstliche Bauchspeicheldrüse, smarte Insuline, individualisierte Therapie	Erklärfilm
Blick in die Diabetesforschung	Einblick in 6 Teilbereiche der Diabetesforschung (Stammzellforschung, Epidemiologie, Immunologie, Systembiologie, Genetik und Epigenetik), Kurzvorstellung der Disziplinen, Bezug zur Diabetesforschung	Interaktive Erklärgrafik

**Table 2:**

Thema	Inhalte	Material/Methode
Grundlagen zum Thema Krebs – Prävention, Entstehung und Behandlung	Wissen, aktuelle Forschung und Präventionsansätze am Beispiel Krebs	Reader für Lehrkräfte, 31 Seiten
Exkurs Krebserkrankung im Schulkontext	Unterstützungsangebote bei Krebserkrankungen im sozialen Umfeld von Schülerinnen und Schülern	Reader für Lehrkräfte, 4 Seiten
Lebensstil	Einführung zu den wichtigsten beeinflussbaren Krebsrisikofaktoren, aber auch zu populären Mythen der Krebsentstehung, einschl. einer Selbstreflexionsübung	Lerneinheit
Übergewicht und Krebs	Normgewicht und Übergewicht – Definitionen, Body-Mass-Index, Energiebilanz, Übergewicht und Krebs: Tumorarten, biologische Prozesse	Lerneinheit mit einem Praxisteil und Selbstreflexionselementen
Bewegung und Krebs	Bewegung, Intensität von körperlicher Bewegung, Konzept des metabolischen Äquivalents	Lerneinheit
Gesunde Ernährung	Eckpfeiler einer gesunden Ernährung; Prinzip des Nudgings in der Schulmensa	Lerneinheit
Humane Papillomviren (HPV) – Eine Einführung	Einführung in das Thema HPV-Impfung anhand eines fiktiven Chats, Vermittlung von Basiswissen zu HPV-induzierten Krankheiten und HPV-Impfung inkl. Lernzielkontrolle für die Klassen 5 bis 7	Lerneinheit
Humane Papillomviren – Von Viren, Sex und Wartezimmern	Viren, HPV und HPV-induzierte Erkrankungen sowie Immunsystem und Impfung inkl. Lernzielkontrolle für die Klassen 7 bis 10	Lerneinheit als Lernzirkel
UV-Strahlung	Zusammensetzung der Sonnenstrahlung, Wirkungen der UV-Strahlung auf den menschlichen Körper, Möglichkeiten zum UV-Schutz	Lerneinheit mit offenem Lernzirkel mit Pflicht- und Wahlstationen
Sunface-App (Dr. Titus Brinker, Heidelberg, Deutschland) – Dein Gesicht der Zukunft	Einsatz einer App zum Thema UV-Belastung im Unterricht, Testung der individuellen Auswirkungen von UV-Strahlung; Onlinefragebogen zur Abschätzung des Hauttyps	Lerneinheit mit App
UV-Strahlung	Erstellung einer Mind-Map zum Krebsrisikofaktor UV-Strahlung (UV-Strahlung, UV-Index, Folgen von UV-Strahlung, Schutz vor UV-Strahlung, Solarien, Hautkrebsfrüherkennung)	Lerneinheit als Gruppenpuzzle
Thirdhand Smoke – Wie schädlich ist kalter Tabakrauch?	Dilemmasituation und Rollenspiel zum Thema Gesundheitsbeeinträchtigung durch Passivrauchen und Tabakrückstände	Lerneinheit
Smokerface und Smokerstopp	Anleitung zum Einsatz von 2 Apps zu den Folgen des Rauchens im Unterricht, Folgen für Haut und Aussehen und für die Gesundheit, Lebenserwartung, finanzielle Situation	Lerneinheit
Neinsagen kann man lernen – Übungen zur Selbstreflexion	Handreichung für einen gesellschaftlich-sozialen Diskurs am Beispiel Rauchen. Durch Selbstreflexion das eigene Handeln in bestimmten Situationen steuern, Umgang mit Statistiken	Lerneinheit
Fermi-Problem: Zigarettenkonsum und Nikotinaufnahme	Komplexe Problemstellungen zum Krebsrisikofaktor Rauchen lösen (vorhandenes Wissen aktivieren, Daten erheben, Schätzungen anstellen, Informationen bewerten)	Lerneinheit
Krebs – Was ist das eigentlich?	Entstehung, Behandlung und Prävention von Krebs, die häufigsten Krebsarten in Deutschland	Lerneinheit
Krebs in Deutschland	Übersicht über Krebsstatistiken, Erstellung eines Vortrags zu einer der 5 häufigsten Krebsarten in Deutschland (z. B. mit der Pecha-Kucha-Vortragstechnik)	Lerneinheit
Kurz erklärt – So entsteht Krebs	Aktivierung von Vorwissen mit der Clustering-Methode, Grundlagen zur Krankheit Krebs	Lerneinheit mit Erklärvideo
Selbst erklärt – So entwickelt sich Krebs	Versprachlichung eines stummen Erklärvideos (Krebsrisikofaktoren, Krebsentstehung und Metastasierung)	Lerneinheit zum Erklärvideo
Familiärer Krebs	Einführung in die Genetik des familiären Brust- und Eierstockkrebs – Einführung der DECIDE-Methode zur strukturierten Entscheidungsfindung bezüglich eines Gentests anhand eines Fallbeispiels	Lerneinheit
Zielgerichtete Krebstherapie	Mystery zur zielgerichteten Krebstherapie, Wirkmechanismen zielgerichteter Medikamente	Lerneinheit
Meilensteine der Krebsforschung	Wichtige Forschungserkenntnisse zu Entstehung, Diagnose, Behandlung und Prävention von Krebs	Lerneinheit mit Edu-Breakouts und Zusatzmaterial, Zeitstrahl

**Table 3:**

Thema	Inhalte	Material/Methode
Durch Medienkompetenz Gesundheit fördern	Einführung in verschiedene Medienformate und Suchmöglichkeiten für Gesundheitsinformationen, Herleitung und Anwendung von Kriterien zur Einschätzung der Qualität von Gesundheitsinformation	Lerneinheit
Gesundheits-Apps	Einführung in verschiedene Arten und Verwendungen von Gesundheits-Apps für den Gebrauch im Alltag. Erkennen guter Apps, Bewerten der Qualität von Messmethoden, Datensicherheit und Finanzierung	Lerneinheit
Analyse der Lehr- und Bildungspläne der allgemeinbildenden Schulen in Deutschland	Diabetes und Krebs	Handbuch für Lehrkräfte, 154 Seiten

## Footnotes

1 Unterrichtsmethode, bei der Schülerinnen und Schüler kooperativ Rätsel lösen. Anhand von Fakten und Indizien auf Rätselkarten erarbeiten sie die Lösung und diskutieren im Anschluss darüber.
